# The impact of subway car interior design on passenger evacuation and boarding/alighting efficiency

**DOI:** 10.1038/s41598-023-47045-4

**Published:** 2023-11-11

**Authors:** Si-jun He, Juan Li, Wen-wen Chen, Tie-cheng Ding, Jin-yi Zhi

**Affiliations:** 1 School of Art and Design, Xihua University, Chengdu, 610039 China; 2 School of Design, Southwest Jiaotong University, Chengdu, 611756 China

**Keywords:** Engineering, Mechanical engineering

## Abstract

This study investigated the impact of subway car interior design on passenger evacuation and boarding/alighting efficiency. The usability of pedestrian agent models was verified through real-life experiments. A seven-factor orthogonal simulation experiment was designed, using key geometric features of the subway car interior as variables. The results of the computer simulation showed that the impact of subway car interior design factors on evacuation and boarding/alighting time was not entirely consistent, with seat layout and door width being the most important factors affecting passenger movement. In the evacuation scenario, only the connectivity of the subway car has no effect on evacuation time, while in the boarding and alighting scenario, seat layout, car type, door width, and foyer width all significantly affect boarding and alighting time. Multivariate regression models were established to predict evacuation and boarding/alighting times through design features, which can explain 86.7% and 58.9% of the time variation, respectively. The research results were used to guide subway car design, and the proposed new scheme demonstrated better performance.

## Introduction

Urban rail transit plays an indispensable role in the social activities of residents. In Seoul, the subway accounts for 65% of the total passenger transportation in the urban public transportation system, while in Shanghai, this ratio reaches 70.31%^1,2^. As a result, the urban rail transit environment in Asian countries is more crowded compared to other regions^3^. Recent research indicates that although the COVID-19 pandemic has made passengers more sensitive to crowded environments in subway cars^4^, passengers still maintain close physical distance between each other^1^. At the subjective level, the estimation of the value of crowding (VoC) is often conducted using Logit models with stated preference (SP) data, and investigates their influence on travel decisions^4–7^. These methods are grounded in the economic perspective and passengers’ static perception of crowding. At the objective level, crowding can lead to a decrease in passenger mobility efficiency, such as increased walking time for access and egress of the train^8^. A limited amount of research has reported on the limitations of crowd movement efficiency within the train due to internal crowding from a safety perspective^9–11^. However, there is still a lack of systematic research on the impact of design factors on the movement of densely populated crowds with a random distribution.

Boarding and alighting are the most basic scenarios of passengers taking rail transit, and higher boarding and alighting efficiency is beneficial for optimizing train operation^12^. Interior design is the key factor affecting boarding and alighting^13^. Importantly, the efficiency of crowd flow inside the train is closely related to passenger evacuation in emergency situations. Many accidents have proven that failure to evacuate the subway car within a limited time may lead to catastrophic consequences, such as the 1995 Azerbaijan metro fire that killed 289 people, the 2003 Daegu subway fire that claimed 192 lives^14^, and the 2021 Zhengzhou subway flooding accident that resulted in 14 deaths and 5 injuries^15^. Most studies on subway evacuation set fire as the main factor for simulating accidents. These studies mainly discuss variables such as smoke concentration^16^, heat release rate^17^, and ignition point^18^. In almost all emergency situations on trains, evacuating passengers is the primary task. Even if not considered in the evacuation scenario, injuries or stampede accidents caused by overcrowding during boarding and alighting occur in 26.67% of cases inside the subway car^19^. Multiple studies have shown that reasonable subway car design plays an important role in improving passenger flow^20–23^. After comparing and reviewing studies on passenger evacuation and boarding/alighting, we found that the geometric features of the subway car have the same physical mechanisms on passenger evacuation and boarding/alighting. However, they are often studied as separate behaviors. Therefore, this study attempted to use the same interior design variables in both evacuation and boarding/alighting processes for experimentation. The objectives of the study are as follows:

- To verify whether the impact of interior design features on evacuation and boarding/alighting is consistent.
- To quantitatively assess the influence of design features on passenger flow efficiency.
- To utilize the research findings to guide train design.

### Literature review

There are many factors can affect passenger movement time, which can be summarized as external factors outside the subway car, factors related to passengers and train staff, and internal factors within the subway car. External factors include station design^24^, platform screen doors (PSD)^25^, vertical and horizontal gaps between trains and platforms^13,21^, and in emergency evacuation scenarios in which the station cannot be reached, the train-side ladders^23^, tunnel exit^26^, etc. are considered. Passenger behavior is also a key factor in causing time differences, such as competition and compromise behavior of passengers during boarding and alighting processes^27^, as well as emergency behavior of passengers and train staff^28–30^. For the interior design of the subway car, Table 1 provides an overview of the research objects, variable settings, methodologies, and main findings of these studies.

**Table 1:** Main literature on the impact of interior design on passenger flow.

Serial no.	Reference	Research object	Method	Data analysis	Interior variables	Findings
1	Seriani and Fernandez^31^	The foyer and platform of the Santiago subway car	LEGION simulation combined with real-life experiments	Descriptive statistics	Position of the pole	A pole close to the door frame is more conducive to passenger flow than a pole in the central of the foyer
2	Neto and Santos^32^	The subway car in Sao Paulo	Real-life experiment on a partial car model	Regression analysis	The number of doors, central pole	The average time for boarding and alighting depends on the total width of the exits, and installing the central pole will take 13% more time
3	Thoreau et al.^21^	The subway car in London	Real-life experiment on a car model	Descriptive statistics	Door width, central pole, seat type	Door width of 1700–1800 mm is best; central pole has no obvious effect on boarding/alighting
4	Fujiyama et al.^33^	The subway car in the UK	Real-life experiment on half of the car model	Descriptive statistics	Door width, foyer size	Increasing the width of the foyer is conducive to passenger flow, but beyond a certain threshold, it does not bring about substantial changes
5	Fridolf et al.^23^	The carriage of X1-type train in Sweden	Real-life experiment on a partial car model	Regression analysis	Light intensity	The absence of lighting inside the train or the presence of emergency stairs at the exits would both significantly reduce the flow rate of people
6	Yu et al.^34^	The train carriage in China	Real-life experiment combined with EXODUS for simulation	Descriptive statistics	Number and location of open doors	It is faster to evacuate by opening two doors on one side of the carriage than opening one door on each side of the carriage
7	Qiu and Fang^22^	The carriage of the CRH5 high-speed train in China	Simulation using LEGION	Regression and variance analysis	Aisle width, door width, seat pitch	The seat pitch and the interaction of the seat pitch and the aisle width impact the evacuation time
8	Schelenz et al.^35^	Two types of virtual design carriages	Simulation using ANYLOGIC	Descriptive statistics	Carriages with three doors or four doors	Compared with three-door carriage, four-door carriage helps to redistribute the passenger flow of the middle door
9	Daamen et al.^13^	The train carriage in Europe	Real-life experiment on the foyer and partial platform models	Descriptive statistics	The vertical and horizontal gaps, door width	Increasing horizontal and vertical gaps can result in a decrease in door capacity by approximately 15%

In the aforementioned study, real-life testing and computer simulation are the most commonly used methods to study passenger flow. Real-life testing allows for the realistic reproduction of passenger behavior during evacuation and boarding/alighting processes^22^. However, due to the high cost involved, partial car models can be used as a substitute for full subway car experiments. When there are many scenarios to be tested in the real-life experiment, considering factors such as scenario replacement and reduced subject physical fitness, the cycle and cost of the experiment are difficult to control, and more importantly, the real-life experiment has unpredictable safety risks. In contrast, computer simulation offers clear advantages in safety, speed, and operating costs. Microscopic pedestrian models can independently describe and calculate the behavior of each person, not only simulating pedestrian traffic flows from a macro perspective but also depicting the complex behavior of pedestrian traffic in detail^36^. The most typical models are the cellular automaton model (CA)^37^, social force model (SFM)^38^, and agent-based model (ABM)^35^, which are widely used for pedestrian flow simulation in various scenarios and have been proven to have minimal differences with real-life experiments^22^.

## Methods

### Real-life experiment

This study established an orthogonal computer simulation scheme of subway cars with different geometric parameters and used ABM to simulate passenger evacuation and boarding/alighting. Before conducting computer simulations, a real-life preliminary experiment was conducted to determine the walking speed at which passengers move within the subway car.

#### Passenger walking speed

The walking speed of passengers is the basis of crowd flow. A train evacuation experiment conducted by the United States Federal Railway Administration (FRA) in Boston shows that average speed for men is 1.5 m/s and for women it is 1.3 m/s^39^. Yu et al.^34^ believed that a walking speed of 1.0–1.2 m/s was reasonable based on evacuation simulations for Chinese trains. According to Luangboriboon et al.^9^, passengers face limited space when boarding, while there is unlimited space to face during evacuations, which may lead to different walking speeds during evacuation and boarding/alighting. Almost all existing studies report walking speeds in the range of 1.0–1.5 m/s. Based on this range, different average speeds can be defined for ABM in computer simulations. By calibrating the simulation to match the time taken in real-life experiments for the same scenario, the walking speed of passengers in real-life scenarios can be determined.

#### Participants

All participants in this study were from Southwest Jiaotong University. A total of 120 participants were recruited for the real-life experiment, including 61 males with an average shoulder width of 44.4 cm, and 59 females with an average shoulder width of 41.5 cm. The participants were between 21 and 32 years old and had experience in taking the subway.

This study was approved by the Ethics Committee of Southwest Jiaotong University and conducted according to the principles of the Declaration of Helsinki. All the participants provided written informed consent before participating. Note that we obtained informed consent from all displayed subjects for the publication of identifying images in an online open-access publication.

#### Agent-based model

The behavior rules of the agents used the Steering model, which combines steering mechanisms and collision handling to control passengers following a curved search path. This model allows passengers to deviate from the path while still moving toward the target direction. Reynolds^40^ and Amor et al.^41^ provide detailed technical information about the Steering algorithm.

#### Materials

The subway car can be simplified into two basic functional modules: the door area and the seating area (including the area in front of the seats). This study built a real-life subway car for experimentation, which replicated one-third of the full-size Chinese wide-body car (type-A), and included the two basic functional modules.

#### Procedures

When 80 participants entered the real-life subway car, the corresponding standing density was 6 pass/m^2^, which is the maximum density for rated passenger capacity in the Chinese subway standard^42^. The areas where standing was not allowed were marked with yellow tape on the floor. Only two extreme scenarios were considered: (1) evacuation of passengers through both doors when the car is fully loaded; (2) with door open on one side, 50% of passengers alight and the same number of passengers board. The two scenarios of the real-life experiment are shown in Table 2.

**Table 2:** Scenario setting of real-life experiment.

Scenario	Passenger behaviour	Door status	Initial number of people in the car	Number of people boarding	Number of people alighting	Number of people stranded in the car	Total number of participants
1	Evacuation	Double-sided opening	80	0	80	0	80
2	Boarding and alighting	Single-sided opening	80	40	40	40	120

**Figure 1:**
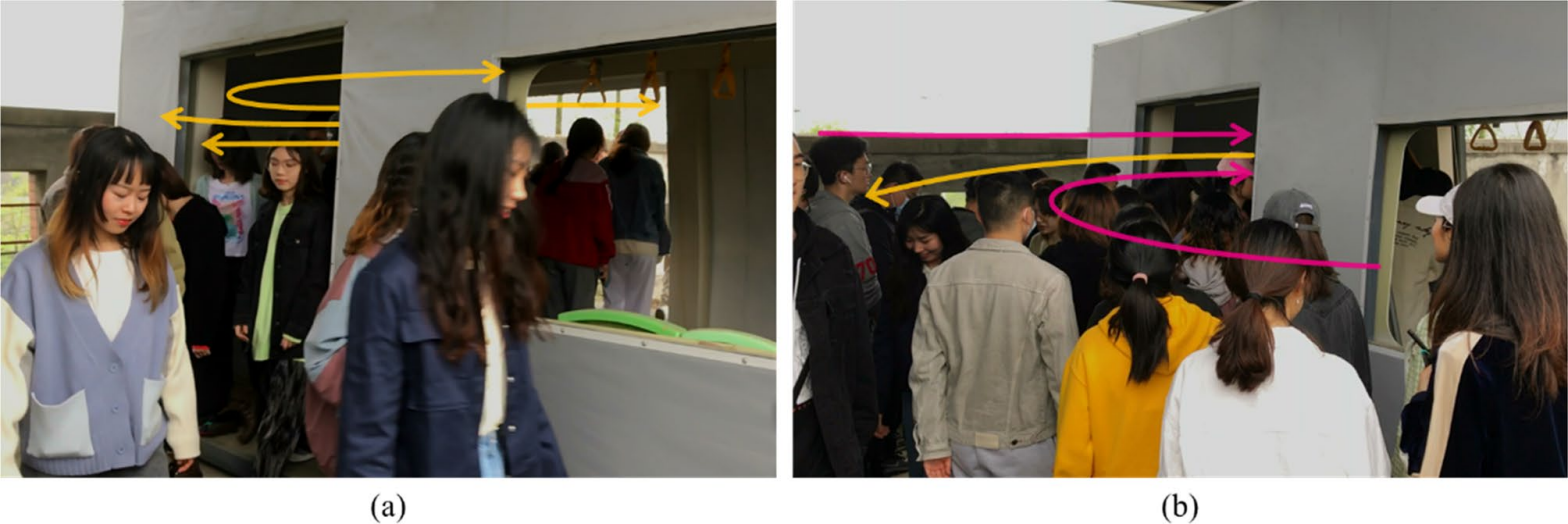
Experiments in real-life scenarios: (**a**) evacuation; (**b**) boarding and alighting.

Each of the two scenarios was tested 10 times in succession (Fig. 1), with a five-minute interval between each test. A dedicated experimenter reported “start” and started timing until the last participant passed through the door, at which point the timing was stopped and the average time for the repeated tests was calculated.

**Figure 2:**
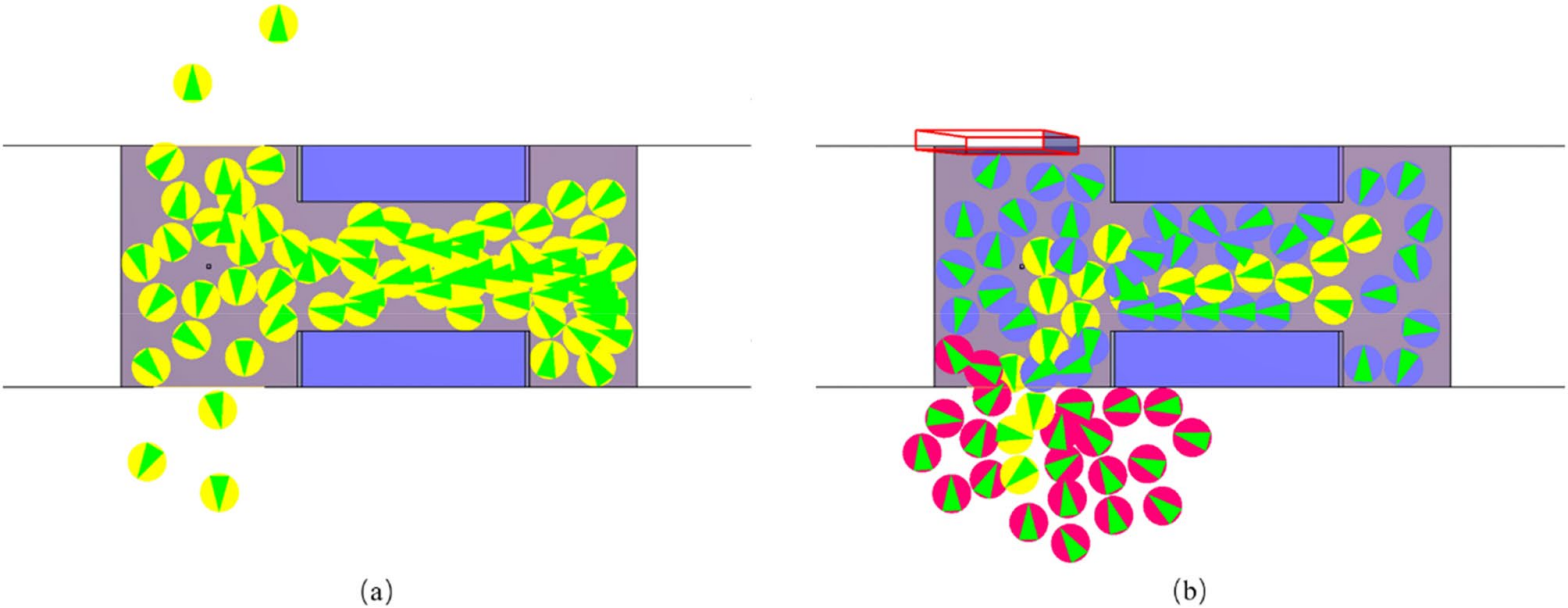
Computer simulations: (**a**) evacuation; (**b**) boarding and alighting, the red, yellow, and blue circles indicate the agents boarding, alighting, and remaining in the car, respectively, and the arrows indicate the direction of movement.

Correspondingly, a digital model identical to the real-life subway car was established, and the gender ratio and average shoulder width of the agents were set in accordance with the participants in the experiment. Simulations of evacuation and boarding/alighting were conducted at speeds of 1.0–1.5 m/s (with intervals of 0.1 m/s). Each simulation scenario at each speed was run 10 times, with the positions of the agents randomly rearranged each time. The simulated scenarios are shown in Fig. 2.

The completion times obtained from the real-life experiment were compared to the completion times obtained from computer simulations at different walking speeds. The results of an independent sample *t*-test showed that there was no significant difference between the completion time of the simulation using a walking speed of 1.4 m/s and the completion time of the real-life evacuation scenario (*p* > 0.05), indicating that a walking speed of 1.4 m/s in the simulation experiment is consistent with the real-life scenario during car evacuation. Similarly, in the boarding and alighting scenario, there was no significant difference between the completion time of the computer simulation using a walking speed of 1.2 m/s and the completion time of the real-life scenario (*p* > 0.05), indicating that a walking speed of 1.2 m/s can be used to simulate the boarding and alighting scenario.

### Computer simulation experiment

The formal experiment was simulated using a computer, with the same passenger behavior settings as the real-life experiment. Agents were added to the digital model at the maximum density of 6 pass/m^2^, using six full-size subway car marshaling models. The occupants were evenly split between males and females, with the maximum shoulder width for the 50th percentile male and female adults set according to the data published by the Human Dimensions of Chinese Adults^43^.

#### Variables

**Figure 3:**
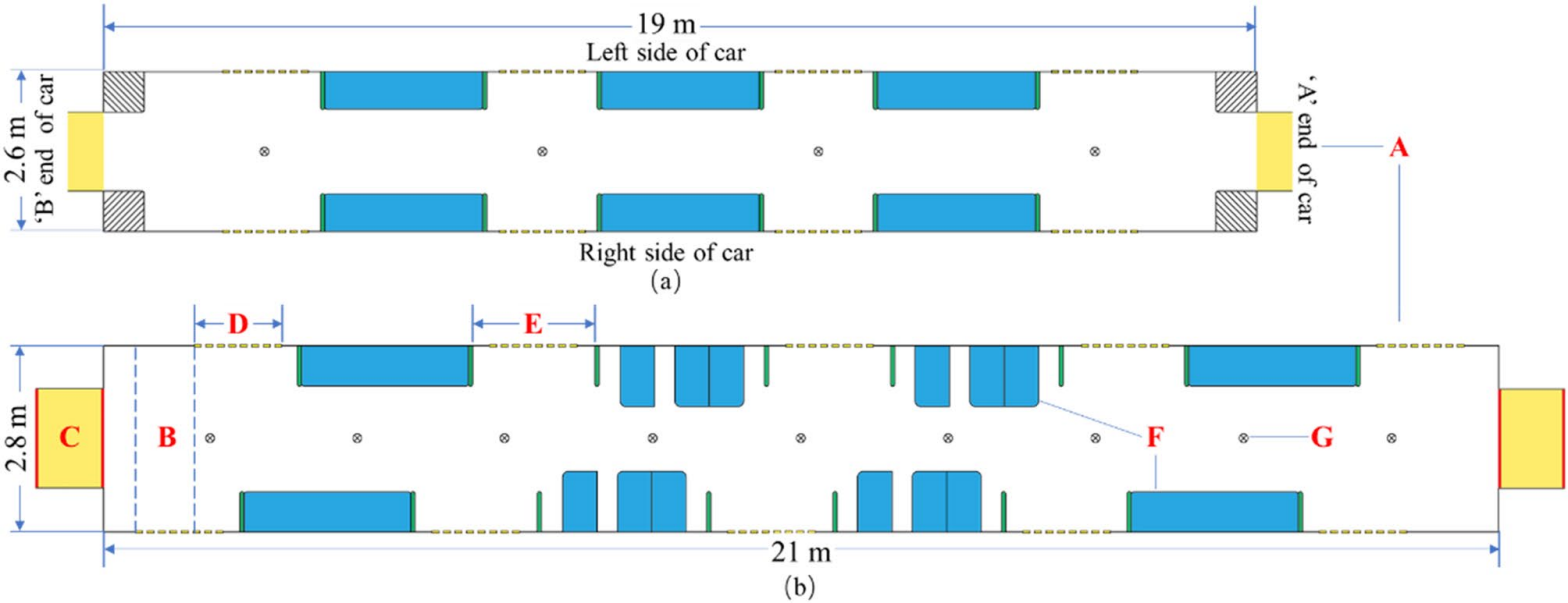
Schematic diagram of variables: (**a**) narrow-body car; (**b**) wide-body car.

Based on the design features of subway cars, the following variables were studied as independent variables: (A) car type, (B) door symmetry, (C) car connectivity, (D) door width, (E) foyer width, (F) seat layout, and (G) pole layout. Figure 3 shows a schematic diagram of the independent variables. Time was used as the dependent variable.

The geometric parameters of these variables, including seat size, pole diameter, and the size of the space at the connection of the car, were consistent with those of the currently operating subway. The Factors and levels of the test are shown in Table 3.

**Table 3:** Factors and levels of the test.

Levels	A	B	C	D	E	F	G
	Type	Door symmetry	Car connectivity	Door width (mm)	Foyer width (mm)	Seat layout	Pole layout
1	Narrow-body	Symmetric	Connected	1300	1650	Longitudinal seat	Without pole
2	Wide-body	Asymmetric	Disconnected	1400	1850	Longitudinal seats only at both ends	One pole in front of seat
3	–	–	–	1500	2050	Transverse and longitudinal alternating seats (opposite and side of longitudinal seats are transverse seats)	One pole in centre of door area
4	–	–	–	–	–	Transverse seats only at both ends	Two poles in front of seat
5	–	–	–	–	–	Only left side at “B” end of car and right side at “B” end of car are longitudinal seats	A pole in each door area and in front of seat
6	–	–	–	–	–	Transverse seats	Two are in front of the seats and one is in the door area

### Orthogonal experimental design

When considering *n* as the number of test scenarios, *r* as the number of levels for each variable, and *m* as the number of variables with *r* levels, the aforementioned full factorial test with two factors at three levels (*r*_1_ = 3, *m*_1_ = 2), three factors at two levels (*r*_2_ = 2, *m*_2_ = 3), and two factors at six levels (*r*_3_ = 6, *m*_3_ = 2) would yield a total of 3^2^ × 2^3^ × 6^2^ = 2592 test scenarios. In order to simplify the testing process, the study plans to establish a mixed-level orthogonal array. According to the characteristics of orthogonal testing, in order to conduct factor analysis on the test results, the number of tests *n* needs to meet a minimum requirement of $n \ge m(r - 1) + 1$. Due to the unequal values of *r* in this study, the minimum requirement for *n* is as follows:

1 $$ n \ge m_{1} (r_{1} - 1) + m_{2} (r_{2} - 1) + m_{3} (r_{3} - 1) + 1 $$

It can be obtained that *n* ≥ 18. In order to satisfy the uniformity and comparability of orthogonal testing, *n* still needs to meet certain constraints:

2 $$ n \ge 18 \cap r \gg \max (r_{1} ,r_{2} ,r_{3} ) \cap \sum {m \ge m_{1} + m_{2} + m_{3} } $$

The experimental table L36 (2^3^ × 3^2^ × 6^2^), can be obtained by solving, which required only 36 schemes to test. To reduce errors, each simulation scenario was run 10 times. The test scheme and the mean value of the results are shown in Table 4.

**Table 4:** Mean and standard deviation of the orthogonal test design.

Test no	A	B	C	D	E	F	G	Average evacuation time (s)		Average boarding and alighting time (s)	
								Mean	SD	Mean	SD
1	1	1	1	1	1	5	5	29.17	1.09	81.15	4.07
2	1	1	1	2	2	1	1	20.13	0.64	73.82	3.30
3	1	1	1	3	3	4	3	20.16	0.70	73.55	4.35
4	1	1	2	1	1	6	6	25.53	0.94	86.72	3.08
5	1	1	2	1	2	3	1	22.77	0.60	81.77	3.76
6	1	1	2	2	2	2	2	21.15	0.57	77.98	3.44
7	1	1	2	2	3	6	3	23.02	1.03	90.10	5.01
8	1	1	2	3	1	2	5	20.41	1.02	71.59	2.87
9	1	1	2	3	3	3	4	21.88	0.69	69.67	3.64
10	1	2	1	1	3	1	5	21.80	0.39	76.36	3.60
11	1	2	1	1	3	6	2	23.97	0.54	80.00	4.33
12	1	2	1	2	1	2	4	21.87	1.02	74.95	3.44
13	1	2	1	2	1	4	1	20.86	0.94	73.69	3.62
14	1	2	1	3	2	3	6	21.08	0.87	72.84	3.34
15	1	2	1	3	2	5	3	20.89	0.55	77.66	3.23
16	1	2	2	1	2	4	2	22.93	0.58	85.45	5.07
17	1	2	2	2	3	5	4	22.55	0.66	76.56	4.89
18	1	2	2	3	1	1	6	20.89	0.61	72.06	4.52
19	2	1	1	1	2	1	4	20.44	0.60	74.03	4.93
20	2	1	1	1	3	2	6	21.96	0.53	77.98	4.31
21	2	1	1	2	1	3	2	19.35	0.41	80.66	5.63
22	2	1	1	2	3	4	6	20.74	0.45	72.03	3.53
23	2	1	1	3	1	5	2	23.88	0.96	88.49	4.24
24	2	1	1	3	2	6	4	19.71	0.33	88.35	4.00
25	2	1	2	1	3	5	1	21.27	0.61	87.09	4.69
26	2	1	2	2	1	1	3	18.40	0.62	66.84	3.19
27	2	1	2	3	2	4	5	19.21	0.80	78.34	4.53
28	2	2	1	1	1	3	3	20.14	0.42	80.79	4.69
29	2	2	1	2	2	6	5	20.41	0.92	84.53	4.98
30	2	2	1	3	3	2	1	18.35	0.29	72.91	2.08
31	2	2	2	1	1	4	4	20.86	1.05	89.45	4.02
32	2	2	2	1	2	2	3	21.50	0.66	83.49	4.41
33	2	2	2	2	2	5	6	20.85	0.60	88.84	4.21
34	2	2	2	2	3	3	5	19.89	0.73	70.08	3.30
35	2	2	2	3	1	6	1	19.67	0.47	79.58	3.06
36	2	2	2	3	3	1	2	17.97	0.69	68.80	2.98

#### Data processing

Descriptive statistics are commonly used to present the results of such studies due to their intuitiveness, but they cannot report the extent of the impact of design features on passenger flow. Using analysis of variance (ANOVA) to analyze orthogonal experimental data is a standard procedure. To confirm the significance of factors on time impact, using the data from each simulation rather than the average values of the scenarios. Post-hoc comparisons were made using the least significant difference (LSD) method, with alpha levels considered significant at 0.05 and very significant at 0.01. Finally, a multiple linear regression (MLR) model was established to predict the evacuation and boarding/alighting times.

## Results

### Factors affecting passenger flow efficiency

**Figure 4:**
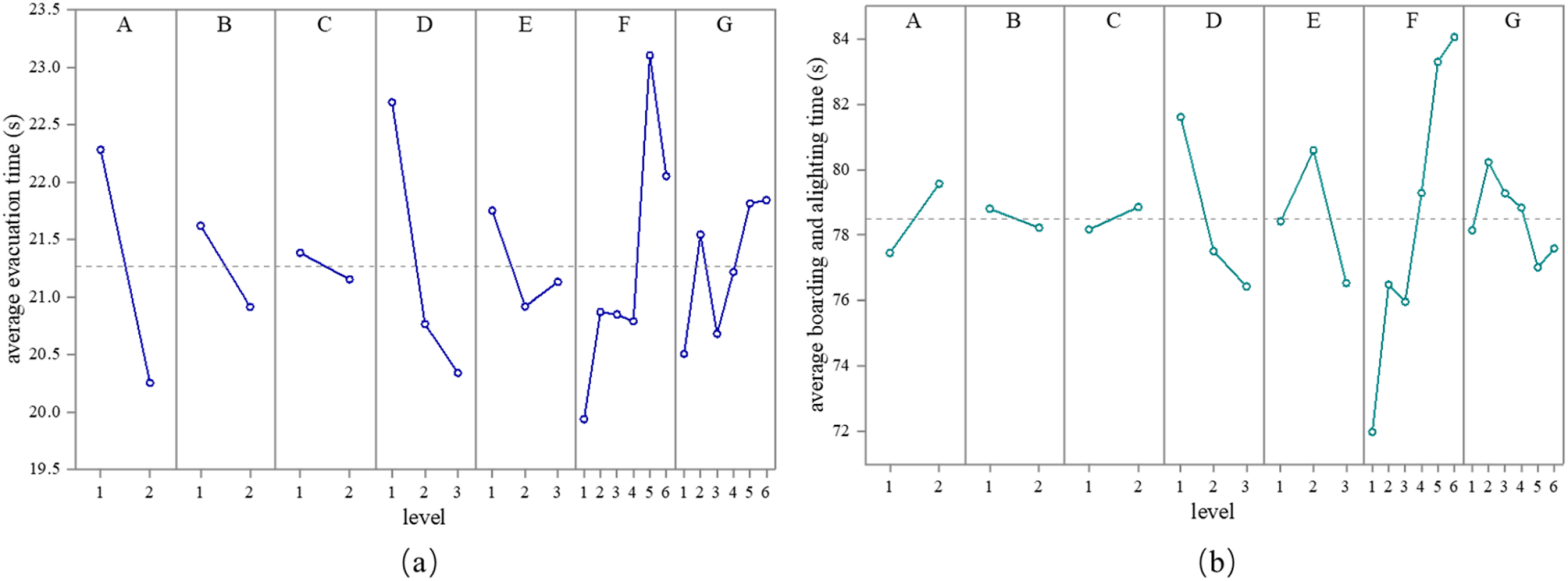
Mean value of the scheme at each level in the factors: (**a**) evacuation time; (**b**) boarding and alighting time.

The level mean value of each factor is shown in Fig. 4, and the results of the ANOVA are listed in Table 5.

**Table 5:** Inter-subject effect.

Factor	Evacuation scenario				Boarding and alighting scenario			
	SS	df	F-value	P-value	SS	df	F-value	P-value
A	369.81	1	294.23	0.000**	401.92	1	6.28	0.013*
B	44.79	1	35.66	0.000**	30.32	1	0.47	0.492
C	4.78	1	3.80	0.052	42.95	1	0.67	0.413
D	377.11	2	150.01	0.000**	1797.44	2	14.03	0.000**
E	44.82	2	17.82	0.000**	991.139	2	7.73	0.001**
F	378.42	5	60.22	0.000**	6450.08	5	20.14	0.000**
G	97.58	5	15.52	0.000**	413.14	5	1.29	0.268

*Represents significance at the level of 0.05.

**Represents significance at the level of 0.01.

The results of the ANOVA indicate that, during passenger evacuation, only car connectivity had a non-significant effect, while during boarding and alighting, car type, door width, foyer width, and seat layout all had a significant effect on efficiency (all *p*-values < 0.05). For the non-significant variables, this means that selecting any level of design parameters for these variables did not have a significant impact on the results.

Wide-body cars were found to be more favorable for evacuation (*p* < 0.001), while narrow-body cars had higher efficiency for boarding and alighting (*p* = 0.013). The use of asymmetrical doors significantly reduced evacuation time (*p* < 0.001), but did not significantly reduce boarding and alighting time. Whether the subway car is connected has no significant effect on evacuation and boarding/alighting time.

The factors of df ≥ 2 are examined via a post hoc test. The results of pairwise comparison show that the difference between any door width was significant during evacuation (all *p*-values < 0.05). In the boarding and alighting scenario, there was no significant difference between the 1400 mm and 1500 mm doors, but both were significantly faster than the 1300 mm doors (all *p*-values < 0.001).

For foyer width, the 1850 mm design performed the best during evacuation, but there was no statistically significant difference from the 2050 mm design. Both the 1850 mm and 2050 mm foyers were faster than the 1650 mm foyer (all *p*-values < 0.001). During boarding and alighting, there was no significant difference between the 1650 mm and 2050 mm foyers, while foyers with a median width of 1850 mm showed a significant disadvantage (all *p*-values < 0.05).

The impact of seat layout on time was highly significant (all *p*-values < 0.001), with all-longitudinal seats taking the least amount of time compared to other layout styles in both evacuation and boarding/alighting scenarios (all *p*-values < 0.001). The mixed layout styles of alternating longitudinal and transverse seats (F3), transverse seats at both ends (F2), and longitudinal seats at both ends (F4) did not have a significant impact on evacuation and boarding/alighting time. However, the design with a set of longitudinal seats at both ends of the car (F5) took the longest time during evacuation (all *p*-values < 0.001), more than the all-transverse seating layout (F6) (*p* < 0.001). During boarding and alighting, there was no difference between level 5 and level 6, but both took significantly more time than other layout styles (all *p*-values < 0.05).

Different pole arrangements only had an impact on passenger evacuation (*p* < 0.001). The shortest evacuation times were observed when no pole was used (G1) or when one pole was installed in the door area (G3), and the difference between the two was not significant. There was little difference between a single pole (G2) and two poles (G4) in front of the seat, but took more time than scenarios with poles only in the door area or not using a pole (all *p*-values < 0.05). The layout with poles installed in both the door area and in front of the seats (G5 and G6) posed the greatest obstacle to evacuation, and there was no significant difference between G5 and G6.

### Predictive model for passenger flow efficiency

One of the important goals of this study was to improve the relevance to train manufacturing. Based on the results of the ANOVA, only significant factors were included in the MLR model, with the first level of all independent variables set as the reference group and the other levels set as dummy variables. The MLR model for predicting evacuation time is as follows:

3 $$ \begin{gathered} Y_{e} = 22.455 - 2.027A2 - 0.706B2 + ( - 1.927D2 - 2.352D3) + ( - 0.831E2 \hfill \\ - 0.621E3) + (0.934F2 + 0.912F3 + 0.853F4 + 3.165F5 + 2.114F6) \hfill \\ + (1.034G2 + 0.175G3 + 0.709G4 + 1.306G5 + 1.335G6) \hfill \\ \end{gathered} $$

Among them, *F*(16, 359) = 64.739, *p* < 0.001, adjusted *R*^2^ = 0.867, indicating that the model is significant and has a good fit, explaining 86.7% of the variation in evacuation time. Similarly, the MLR model to predict boarding and alighting time is as follows:

4 $$ \begin{gathered} Y_{b} = 73.931 + 2.113A2 + ( - 4.106D2 - 5.187D3) + (2.172E2 - 1.889E3) \hfill \\ + (4.498F2 + 3.982F3 + 7.308F4 + 11.316F5 + 12.074F6) \hfill \\ \end{gathered} $$

Among them, *F*(10, 359) = 15.027, *p* < 0.001, with an adjusted *R*^2^ = 0.589, indicating that the model is significant and has moderate goodness of fit, explaining 58.9% of the variance in the boarding and alighting time.

## Discussion

This study compared narrow-body subway cars with four-door pairs and wide-body cars with five-door pairs, but there was no consistent conclusion as to which performed better in evacuation and boarding/alighting. Wide-body cars were found to be more conducive to passenger evacuation, as the increased number of doors meant a greater total exit width, consistent with the conclusion of Yu et al.^34^ In addition, wide-body cars have wider aisles, which Qiu and Fang^22^ suggest is an auxiliary factor that facilitates evacuation. Conversely, narrow-body subway cars are more conducive to boarding and alighting, as passengers have limited space for movement after boarding, thereby shortening the time for passenger flow exchange. The effect of car type on evacuation was significant at the *α* = 0.001 level, whereas the effect on boarding and alighting was only significant at the *α* = 0.05 level. Therefore, wider cars are encouraged, especially from a safety perspective.

All of the current subway cars in China are symmetrical in design. Berkovich et al.^44^ argue that the symmetrical layout of doors will cause passengers to crowd around the doors and increase the load in these areas. Asymmetric layouts are a novel design concept, and considering their widespread use in New York and other cities (such as the R-142 and R-32 models manufactured by Bombardier Transportation), it is necessary to explore the potential application of asymmetric designs from the perspective of passenger flow. The results of the study confirmed the superiority of asymmetric designs in both evacuation and boarding/alighting. For train manufacturers, asymmetric designs do not increase the cost of production or the construction of platforms.

Although the connectivity of subway cars does not affect the efficiency of passenger flow, connected cars are more conducive to the evacuation of passengers to adjacent cars in a fire^34^. Passenger circulation across cars also helps to alleviate congestion in individual cars and improve train utilization. Overall, there are more benefits of using connected cars are greater.

Door width plays a crucial role in both evacuation and boarding/alighting. Some viewpoints suggest that when the exit width increases to a certain point, the improvement in the efficiency of personnel flow will become increasingly limited^45^. In evacuation scenarios, when the door width increases from 1400 to 1500 mm, the reduction in evacuation time becomes slower. This trend is more pronounced in boarding and alighting scenarios, where there is no difference in boarding and alighting time between doors with widths of 1400 mm and 1500 mm. Therefore, it is recommended to set a door width of at least 1400 mm.

An increase in foyer width does not always correspond to a decrease in time. The design with a medium-width foyer (1850 mm) is the most controversial, as it performs best in evacuation but has obvious disadvantages in boarding and alighting. Thoreau et al.^21^ revealed a nonlinear relationship between foyer width and flow efficiency of passengers, while Fujiyama et al.^33^ believed that an overly wide foyer creates space that allows passengers to linger, thereby leading to limited boarding and alighting benefits, as the distance between the door frame and the seat partition increases. Parameters that perform extremely poorly in any aspect will not be considered in the design, so a foyer width of 2050 mm performs best in terms of overall performance.

Seat layouts used in almost all active subways are considered in this study. The longitudinal seat layout provides more standing space during peak periods and is therefore the most widely used. Longitudinal seat leaves the widest aisle, which is beneficial for evacuation and boarding/alighting. In recent years, many cities have begun to purchase mixed-layout subway cars, which provide the same number of seats as longitudinal layouts but are far less efficient in terms of evacuation and boarding/alighting. Moreover, the more horizontal seats there are and the more disorderly the layout, the greater the hindrance to crowd flow.

In previous studies, it has been controversial whether a pole at the door area affects the flow of people. Neto and Santos^32^ believe that poles in the door area hinder passengers entering and exiting, while Seriani and Fernandez^31^ have found that poles in the foyer can play a role in diversion. Thoreau et al.^21^ believe that the presence or absence of poles in the door area has no effect on passenger flow. This study did not find any significant impact of poles located on the central axis of the car on boarding and alighting, and while the best evacuation results were achieved without poles, the design of one pole in the door area (G3) should be considered first, taking into account the need to balance service demands.

**Figure 5:**
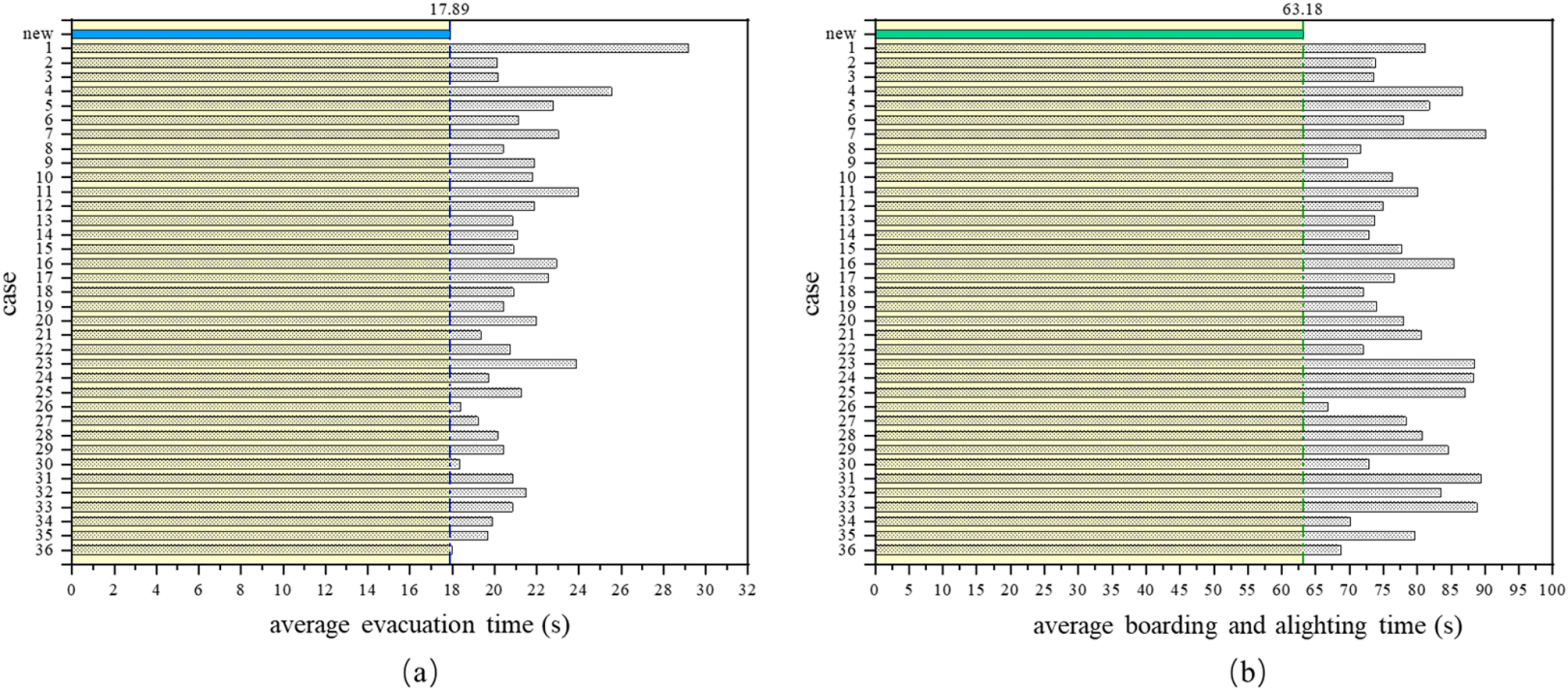
Performance of new scheme in evacuation and boarding/alighting scenarios: (**a**) evacuation; (**b**) boarding and alighting.

With the subway as the public transportation mode with the highest capacity, any small improvement in efficiency at any given time will increase the marginal utility of operational organization and safety. Based on the discussions above, a theoretical parameter combination could be considered: wide-body car (A2), asymmetric doors (B2), connected cars (C1), 1500 mm door width (D3), 2050 mm foyer width (E3), longitudinal seat (F1), and one pole in the door area (G3). This new combination of car design parameters was not included in the typical scheme of orthogonal experiments, which is a good indication. By predicting through the MLR models of Eqs. (1) and (2), the predicted evacuation time for the new scheme is 16.92 s, and the predicted boarding and alighting time is 68.96 s. To verify the accuracy of the prediction model, a digital model was established to simulate this new scheme. The simulation result showed an evacuation time of 17.89 s and a boarding and alighting time of 63.18 s. Compared with the 36 experimental schemes, the new design has the shortest time (Fig. 5) and is not far from the prediction result of the MLR model. To better illustrate the actual changes brought by the new scheme, the ratio of the total number of people passing through the doors to the time is used to represent the efficiency per unit time. In the evacuation simulation, the number of people escaping per second is 93.16, while in the boarding and alighting simulation, it is 27.16 people per second. In subway trains that operate at intervals of 2–5 min each day, even a few seconds of reduction in boarding and alighting time is translated into significant overall time gains.

## Conclusion

This study investigated the impact of design features inside the subway car on passenger evacuation and boarding/alighting efficiency. A mixed-level orthogonal test of seven car design factors was designed, and the results of pedestrian dynamics simulation showed that the impact of design features on evacuation and boarding/alighting was not entirely consistent. Six factors had a significant impact on evacuation, while only four factors had an impact on boarding and alighting. Seat layout and door width were the two most important factors affecting passenger flow efficiency. A MLR model for predicting time was established using significant factors. An optimal parameter layout scheme that comprehensively considered evacuation and boarding/alighting was proposed and verified through the prediction model and computer simulation.

However, the experiment also had certain limitations. Since the purpose of this study was to improve subway cars, other influencing factors were reduced as much as possible, such as disruptions caused by the elderly, disabled people, and pregnant women to the flow of people. and mainly the geometric features of the subway car were discussed. In reality, there are many factors that affect passenger flow efficiency, especially during boarding and alighting. The model for predicting boarding and alighting time had only 0.589 goodness of fit, which also reflects the complexity of this behavior. Future research will further consider these conditions, such as platform evacuation methods with one-sided door opening and transfer methods with boarding on one side and alighting on the other.

## Data Availability

The data that support the findings of this study are available on request from the corresponding author/s. The data are not publicly available due to privacy or ethical restrictions.

## References

[1] Yun, H., Lee, E. H., Moon, S., & Kim, D. K. Data-driven approach for measuring and managing physical distancing in subways during pandemic conditions. *Transport. Res. Rec*. 03611981231190394. 10.1177/03611981231190394 (2023).

[2] China Association of Metros. Urban rail transit 2022 annual statistical and analysis report. *China Metros*. **4**, 13–15. 10.14052/j.cnki.china.metros.2023.04.002 (2023).

[3] Shin H, Kim DK, Kho SY, Cho SH (2021). Valuation of metro crowding considering heterogeneity of route choice behaviors. Transport. Res. Rec..

[4] Singh J, De Almeida Correia GH, Van Wee B, Barbour N (2023). Change in departure time for a train trip to avoid crowding during the COVID-19 pandemic: A latent class study in the Netherlands. Transport. Res. A-Pol..

[5] Li Z, Hensher DA (2011). Crowding and public transport: A review of willingness to pay evidence and its relevance in project appraisal. Transp. Policy..

[6] Shelat S, Cats O, Van Cranenburgh S (2022). Traveller behaviour in public transport in the early stages of the COVID-19 pandemic in the Netherlands. Transport. Res. A-Pol..

[7] Hörcher D, Graham DJ, Anderson RJ (2017). Crowding cost estimation with large scale smart card and vehicle location data. Transport. Res. B-Meth..

[8] Lee EH, Kim K, Kho SY, Kim DK, Cho SH (2022). Exploring for route preferences of subway passengers using smart card and train log data. J. Adv. Transport..

[9] Luangboriboon N, Seriani S, Fujiyama T (2021). The influence of the density inside a train carriage on passenger boarding rate. Int. J. Rail Transp..

[10] Han X, Ma JN, Cong BH (2012). Simulation analysis on crowd evacuation of the subway train fire. Adv. Mat. Res..

[11] Zou Q, Fernandes DS, Chen S (2021). Agent-based evacuation simulation from subway train and platform. J. Transp. Saf. Secur..

[12] Wang S, Zhang W, Qu X (2018). Trial-and-error train fare design scheme for addressing boarding/alighting congestion at CBD stations. Transport. Res. B-Meth..

[13] Daamen W, Lee YC, Wiggenraad P (2008). Boarding and alighting experiments: Overview of setup and performance and some preliminary results. Transport. Res. Rec..

[14] Jeon G, Hong W (2009). Characteristic features of the behavior and perception of evacuees from the Daegu subway fire and safety measures in an underground fire. J. Asian Archit. Build..

[15] Carvel, R., & Marlair, G. *A History of Fire Incidents in Tunnels* (second ed.) 3–23 (ICE Publishing, 2005).

[16] Zhilei W, Min H, Dayong X, Xuhai P (2014). Simulation research on human evacuation in subway with a single-point fire scenario. Proc. Eng..

[17] Ng YW, Chow WK, Cheng CH, Chow CL (2019). Scale modeling study on flame colour in a ventilation-limited train car pool fire. Tunn. Undergr. Sp. Tech..

[18] Lönnermark A, Ingason H, Li YZ, Kumm M (2017). Fire development in a 1/3 train carriage mock-up. Fire Saf. J..

[19] Yu H, Wang Y, Qiu P, Chen J (2019). Analysis of natural and man-made accidents happened in subway stations and trains: Based on statistics of accident cases. MATEC Web. Conf..

[20] Coxon, S., Burns, K., de Bono, A., & Napper, R. An examination of three approaches to metro rolling stock design to ameliorate extended dwell times due to passenger growth and associated crowding. In *34th Australasian Transport Research Forum (ATRF) Proceedings* (2011).

[21] Thoreau R (2016). Train design features affecting boarding and alighting of passengers. J. Adv. Transport..

[22] Qiu H, Fang W (2019). Effect of high-speed train interior space on passenger evacuation using simulation methods. Phys. A.

[23] Fridolf K, Nilsson D, Frantzich H (2014). The flow rate of people during train evacuation in rail tunnels: Effects of different train exit configurations. Saf. Sci..

[24] Cheng H, Yang X (2012). Emergency evacuation capacity of subway stations. Proc. Soc. Behav. Sci..

[25] Qu L, Chow WK (2012). Platform screen doors on emergency evacuation in underground railway stations. Tunn. Undergr. Sp. Tech..

[26] Fridolf K, Nilsson D, Frantzich H (2016). Evacuation of a metro train in an underground rail transportation system: Flow rate capacity of train exits, tunnel walking speeds and exit choice. Fire Technol..

[27] Zhang Q, Han B, Li D (2008). Modeling and simulation of passenger alighting and boarding movement in Beijing metro stations. Transport. Res. C-Emer..

[28] Philpot R, Levine M (2022). Evacuation behavior in a subway train emergency: A video-based analysis. Environ. Behav..

[29] Shiwakoti N, Tay R, Stasinopoulos P, Woolley PJ (2017). Likely behaviors of passengers under emergency evacuation in train station. Saf. Sci..

[30] Dell'Olio L, Ibeas A, Barreda R, Sañudo R (2013). Passenger behavior in trains during emergency situations. J. Saf. Res..

[31] Seriani S, Fernandez R (2015). Pedestrian traffic management of boarding and alighting in metro stations. Transport. Res. C-Emer..

[32] Neto PLDO, Santos CMDD (2002). Ergonomical and statistical aspects in the design of a subway carriage. Gestao Prod..

[33] Fujiyama T, Thoreau R, Tyler N (2014). The effects of the design factors of the train-platform interface on pedestrian flow rates. Pedes. Evac. Dyn..

[34] Yu L, Deng T, Wang MN, Li Q, Xu SS (2019). Passengers’ evacuation from a fire train in railway tunnel. Int. J. Rail Transp..

[35] Schelenz T, Suescun Á, Wikström L, Karlsson M (2014). Application of agent based simulation for evaluating a bus layout design from passengers’ perspective. Transport. Res. C-Emer..

[36] Hu M, Shi Q (2009). Comparative study of pedestrian simulation model and related software. J. Transp. Saf. Secur..

[37] Blue VJ, Adler JL (2001). Cellular automata microsimulation for modeling bi-directional pedestrian walkways. Transport. Res. B-Meth..

[38] Helbing D, Molnar P (1995). Social force model for pedestrian dynamics. Phys. Rev. E..

[39] Markos, S. H., & Pollard, J. K. Passenger train emergency systems: Single-level commuter rail car egress experiments. In *Prepared by Volpe Center/USDOT for FRA/USDOT.* DOT/FRA/ORD-15/04. https://rosap.ntl.bts.gov/view/dot/12181/dot_12181_DS1.pdf (2015).

[40] Reynolds, C. W. Steering behaviors for autonomous characters. In *Proceedings of the Game Developers Conference*. **1999,** 763–782. http://www.red3d.com/cwr/steer/gdc99 (1999).

[41] Amor, H. B., Obst, O., & Murray, J. *Fast, neat and under control: Inverse steering behaviors for physical autonomous agents* (University of Koblenz and Landau, 2003).

[42] GB 50157–2013. *Code of design metro 2013*. Ministry of Construction of the People’s Republic of China, Beijing.

[43] GB 10000–88. *Human dimensions of Chinese adults 1988*. The State Bureau of Quality and Technical Supervision, Beijing.

[44] Berkovich A, Lu A, Levine B, Reddy AV (2013). Observed customer seating and standing behavior and seat preferences on board subway cars in New York City. Transport. Res. Rec..

[45] Song X, Ma L, Ma Y, Yang C, Ji H (2016). Selfishness-and selflessness-based models of pedestrian room evacuation. Phys. A.

